# Regulatory Characterization of Two Cop Systems for Copper Resistance in *Pseudomonas putida*

**DOI:** 10.3390/ijms26178172

**Published:** 2025-08-22

**Authors:** Huizhong Liu, Yafeng Song, Ping Yang, Qian Wang, Ping Huang, Zhiqing Zhang, Gang Zhou, Qingshan Shi, Xiaobao Xie

**Affiliations:** 1 Guangdong Provincial Key Laboratory of Microbial Culture Collection and Application, State Key Laboratory of Applied Microbiology Southern China, Institute of Microbiology, Guangdong Academy of Sciences, Guangzhou 510070, China; liuhuizhong@gdim.cn (H.L.); songyf@gdim.cn (Y.S.); yangping@gdim.cn (P.Y.); wangqian@gdim.cn (Q.W.); huangping@gdim.cn (P.H.); zhangzhiqing@gdim.cn (Z.Z.); zgbees@gdim.cn (G.Z.); jigan@gdim.cn (Q.S.); 2 Guangdong Detection Center of Microbiology, Guangzhou 510070, China

**Keywords:** *Pseudomonas putida*, two-component system, Cop system, copper resistance, regulation analysis

## Abstract

Copper ions serve as essential cofactors for many enzymes but exhibit toxicity at elevated concentrations. In Gram-negative bacteria, the Cop system, typically encoded by *copABCD*, plays a crucial role in maintaining copper homeostasis and detoxification. The chromosome of *Pseudomonas putida* harbors two *copAB* clusters but lacks *copCD*, along with two *copR*-*copS* clusters that encode the cognate two-component system. Here, the roles of these Cop components in countering copper toxicity were studied. We found that *copAB2* was essential for full resistance to Cu^2+^ in *P. putida*, while *copAB1* made only a minor contribution, partially due to its low expression. The two-component systems CopRS1 and CopRS2 both played significant regulatory roles in copper resistance. Although they could compensate for the absence of each other to mediate copper resistance, they exhibited distinct regulatory effects. CopR1 bound to all four *cop* promoters and activated their transcription under copper stress. In contrast, though CopR2 bound to the same sites as CopR1 in each *cop* promoter, it significantly activated only *copAB2* and *copRS2* expression. Its competitive binding at the *copAB1* and *copRS1* promoters likely impeded CopR1-mediated activation of these genes. Overall, this study reveals the distinct contributions of the two Cop systems to copper resistance and their regulatory interplay in *P. putida*.

## 1. Introduction

In biological systems, copper (Cu) is an essential trace element that serves as a versatile cofactor for many redox-active enzymes involved in fundamental metabolic processes [1,2]. Due to the low solubility of cuprous compounds and the instability of free aqueous Cu^+^ ions, the copper that organisms come into contact with in the environment is usually in its oxidized Cu^2+^ state. Cellular acquisition of copper ions typically occurs through transmembrane transport mechanisms involving either passive diffusion via porin channels or active energy-dependent transporters [3]. Current evidence suggests that the outer-membrane porin OmpF/OpmC and the TonB-dependent transporter NosA/OprC might be responsible for the majority of copper imports into the periplasmic space [3,4,5]. Subsequent translocation of the periplasmic copper to the cytoplasm requires major-facilitator superfamily (MFS) transporters, notably CcoA homologs, and other uncharacterized transport systems [3,6]. This essential micronutrient exhibits cytotoxicity at elevated concentrations, as it can generate reactive oxygen species, interfere with the interaction between proteins, and affect the intracellular homeostasis of other metal ions [7,8]. Cells need to tightly control the concentration of intracellular free copper within a narrow physiological range.

The effective strategy for bacteria to maintain the essential copper homeostasis for survival is to export excess copper ions from the cytoplasm to the periplasm or extracellular space. This process is typically associated with P1B-type ATPase transporters, resistance-nodulation-cell division (RND) family efflux pumps, and multicopper oxidases [3,9]. Within the cytoplasm, Cu^2+^ undergoes reduction to its monovalent form (Cu^+^) mediated by nonspecific metalloreductases and endogenous chemical reductants such as glutathione and ascorbic acid [3,10]. The intracellular Cu^+^ is generally bound by copper chaperones and Cu^+^-sensing transcriptional regulators, suggesting that free Cu^+^ is unlikely to exist within cells [11,12]. Cytoplasmic copper chaperones such as CopZ deliver Cu^+^ to the P1B-type ATPase transporters. These inner-membrane transporters subsequently export Cu^+^ to the periplasmic space, where multi-copper oxidases like CueO and PcoA catalyze the oxidation of Cu^+^ to less toxic Cu^2+^ [13,14,15]. Moreover, cytoplasmic and the periplasmic Cu^+^ can be transported to the extracellular space by the RND family transporter CusCBA, which spans from the inner membrane to the outer membrane, with the assistance of the periplasmic copper chaperone CusF [16,17]. However, the significant contribution of CusCBA to copper resistance was observed specifically under O_2_-limiting conditions in *Escherichia coli* [18]. The copper efflux by CusCBA was found to be enhanced by the periplasmic protein CopG, as it can reduce Cu^2+^ into the substrate Cu^+^ for this exporter under anaerobic conditions [19]. Another well-known copper defense system in Gram-negative bacteria is the Cop/Pco systems, which are encoded by *copABCD* or *pcoABCDE* in different bacterial strains [20,21,22]. The multi-copper oxidase CopA/PcoA is responsible for catalyzing the oxidation of periplasmic Cu^+^ to Cu^2+^ [3,23]. The outer membrane protein CopB/PcoB is a transporter that allows Cu^2+^ to enter the periplasmic space from the extracellular environment, and it may also be involved in sequestering Cu^2+^ under copper stress [24]. CopC/PcoC and PcoE are periplasmic proteins that bind copper ions, and CopD/PcoD is a proposed copper permease involved in copper uptake across the inner membrane [25]. Their role in copper management is uncertain. Overall, the transportation and redox activity of copper are complex processes that depend on coordinated interactions between multiple systems, and these are essential for maintaining copper homeostasis within cells.

Various regulators have been employed by bacteria to control the genes encoding different copper defense systems [26]. The MerR-type regulator CueR is a well-studied one-component activator that controls the transcription of genes encoding multi-copper oxidase (CueO) and Cu^+^ transporting P1B-type ATPase [27,28]. CueR exhibits an extremely high affinity for Cu^+^, allowing it to receive Cu^+^ from the cytoplasmic copper chaperone CopZ [12,29]. Structural analysis reveals that CueR dimer reduces the distance between the −10 and −35 elements by bending DNA, thereby facilitating the binding of σ^70^-RNAP holoenzyme to the promoter and activating the transcription [30]. On the other hand, the Cus and Cop/Pco systems are controlled by the two-component systems CusRS and CopRS/PcoRS, respectively [31,32,33]. Upon binding to Cu^+^ in the periplasmic space, the histidine kinase CusS undergoes autophosphorylation, followed by transfer of the phosphate group to the cognate response regulator CusR. The phosphorylated CusR is able to activate the transcription of its target genes [34]. Unlike the typical histidine kinase, CopS in *Pseudomonas aeruginosa* exhibits phosphatase activity towards phosphorylated CopR when the periplasmic copper level is low. Once CopS binds to copper ions, its phosphatase activity is turned off, thereby allowing CopR to retain phosphorylation and activate gene transcription [35]. These systems collectively regulate the copper homeostasis; however, there is still a lack of understanding of the coordinated work of these independent systems.

**Figure 1:**
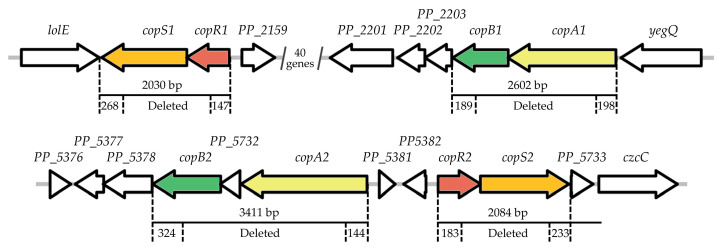
Schematic diagram of the copAB and copRS gene clusters and their deleted regions in the cop mutants.

*Pseudomonas putida* is a Gram-negative bacterium that is commonly found in the environment and exhibits remarkable adaptability to various environmental pressures. One of the best-characterized strains in this species is *P. putida* KT2440, which is a plasmid-free derivative of the soil-isolated strain *P. putida* mt-2 [36,37]. Genomic analysis revealed that its chromosome harbors two *copAB* and two *copRS* gene clusters associated with the Cop system (Figure 1) while notably lacking identifiable *copC* or *copD* homologs [38]. This prompted us to study the roles of different *copAB* and *copRS* operons in copper resistance. Evidence was also provided to show that two CopRs exhibited differential regulatory effects on the expression of *cop* clusters, although both recognize identical binding sites in their target promoters.

## 2. Results

### 2.1. Roles of copAB1 and copAB2 Clusters in Copper Resistance

The genome of *P. putida* KT2440 (NC_002947.4) harbors two gene clusters encoding CopA and CopB proteins. Sequence analysis revealed that the periplasmic multi-copper oxidase CopA1 (PP_2205) shares 62% identity and 71% similarity with CopA2 (PP_5380), while the outer membrane transporter CopB1 (PP_2204) exhibits 56% identity and 72% similarity to CopB2 (PP_5379). It is also noteworthy that CopA2/CopB2 exhibits a higher amino acid sequence identity to PcoA/PcoB from both *E. coli* and *P. aeruginosa* than CopA1/CopB1 does (Table S1). Moreover, compared to CopA1, CopA2 contains more repetitions of the MXXMXHXXM (MDH) motif (Figure S1A), which is implicated in copper binding [38,39]. Interestingly, the MDH motif was also identified in CopB2 but not in CopB1 (Figure S1B). Additionally, unlike the close linkage between *copA1* and *copB1*, a small intervening gene (*PP_5732*) is present between *copA2* and *copB2* (Figure 1). This gene is predicted to encode a metal-binding protein; however, its function remains unknown. These observations suggest that *copAB1* and *copAB2* might play distinct roles in copper resistance.

**Figure 2:**
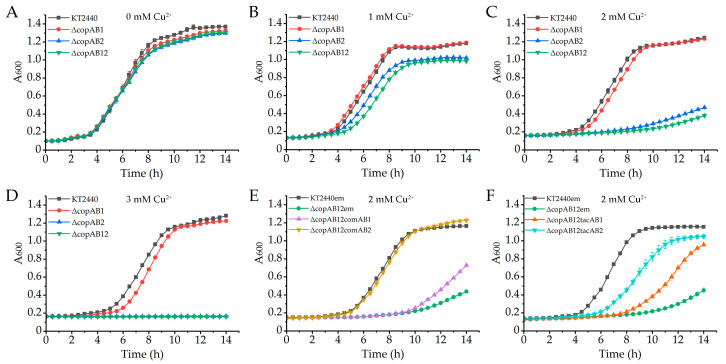
Contribution of *copAB* to copper resistance. *P. putida* strains were cultured in LB medium supplemented with indicated concentrations of CuSO_4_ (**A**–**F**). (**E**) Wild-type and Δ*copAB12* strains harboring either empty pBBR1MCS-5 (em) or pBBR1-*copAB1* (comAB1) and pBBR1-*copAB2* (comAB2). (**F**) Wild-type and Δ*copAB12* strains harboring either empty pBBR1B403 (em) or pB403-*copAB1* (tacAB1) and pB403-*copAB2* (tacAB2). The data represent the mean ± standard deviation of three replicates.

To elucidate the contributions of *copAB* clusters to copper resistance, we generated *P. putida* mutant strains with single or double knockouts of these gene clusters (Figure 1). The copper resistance of the wild-type and mutant strains was assessed by monitoring their growth in Luria–Bertani (LB) medium supplemented with CuSO_4_. Deletion of *copAB1* did not significantly affect growth under Cu^2+^ stress at concentrations below 3 mM (Figure 2A–D). In contrast, knockout of *copAB2* caused growth arrest in the presence of 3 mM Cu^2+^, and even 1 mM Cu^2+^ exerted a slight inhibitory effect on the mutant (Figure 2B,D). The double mutant Δ*copAB12* displayed a copper sensitivity phenotype similar to that of Δ*copAB2* (Figure 2A–D). Complementation experiments revealed that expression of *copAB1* under its native promoter only marginally improved the growth of Δ*copAB12* under 2 mM Cu^2+^ conditions, whereas ectopic expression of *copAB2* fully restored copper resistance to the wild-type levels (Figure 2E). These findings demonstrate that *copAB2* but not *copAB1* plays a critical role in conferring copper resistance in *P. putida* KT2440. To ensure equal expression levels, both *copAB* clusters were expressed under the control of the inducible *tac* promoter. Under these conditions, *copAB1* partially restored the growth of Δ*copAB12* in 2 mM Cu^2+^, although its effect was weaker than that of *copAB2* (Figure 2F). Additionally, *copAB2* exhibited greater copper resistance under its native promoter than under the *tac* promoter (Figure 2E,F), likely due to the higher expression level driven by its native promoter. Collectively, these results suggest that CopAB2 contributes more significantly to copper resistance than CopAB1, potentially due in part to their differential expression levels.

### 2.2. Functional Redundancy of CopRS1 and CopRS2 in Copper Resistance Regulation

**Figure 3:**
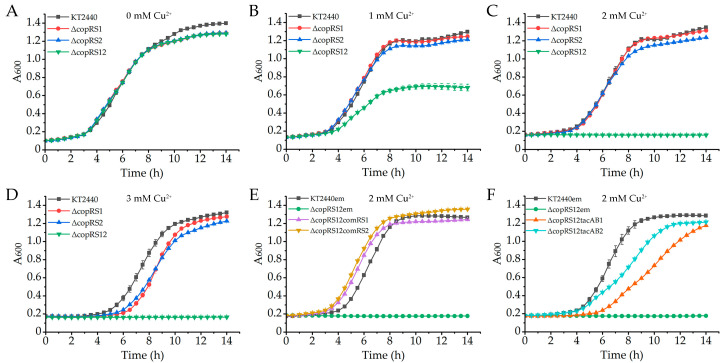
Roles of *copRS* in copper resistance. Growth of *P. putida* KT2440 wild-type and mutant strains was detected in LB medium supplemented with CuSO_4_ (**A**–**F**). (**E**) The wild-type and Δ*copRS12* strains harboring either empty pBBR1MCS-5 (em) or pBBR1-*copRS1* (comRS1) and pBBR1-*copRS2* (comRS2). (**F**) Wild-type and Δ*copRS12* strains harboring either empty pBBR1B403 (em) or pB403-*copAB1* (tacAB1) and pB403-*copAB2* (tacAB2). The data represent the mean ± standard deviation of three replicates.

Two CopRS two-component systems in *P. putida* KT2440 are encoded by *PP_2158*/*PP_2157* (*copR1*/*copS1*) and *PP_5383*/*PP_5384* (*copR2*/*copS2*). The response regulator CopR1 shares 70% identity and 82% similarity with CopR2 at the amino acid level, and the histidine kinase CopS1 shares 43% identity and 59% similarity with CopS2 (Figure S1C,D). Growth detection demonstrated that individual deletion of either *copRS* cluster caused no growth defect under stress of 2 mM Cu^2+^, whereas the double mutant Δ*copRS12* displayed significantly impaired copper resistance (Figure 3A–D). Complementation experiments further confirmed this functional redundancy, as expression of either *copRS1* or *copRS2* from plasmid restored wild-type-level copper resistance in Δ*copRS12* (Figure 3E). This functional equivalence extends to downstream regulatory targets. Previous studies have established that CopRS systems activate transcription of genes involved in copper homeostasis, including their own coding genes [28,40]. To test their regulatory relationship with the *copAB* clusters, we introduced *copAB1* and *copAB2* under the control of the *tac* promoter into the Δ*copRS12* background. Ectopic expression of either *copAB* cluster was able to increase the copper resistance of the mutant (Figure 3F), suggesting that the CopRS systems mediate copper resistance partially through transcriptional activation of both *copAB* clusters.

### 2.3. Regulation of Cop Genes by the CopRS Two-Component Systems

**Figure 4:**
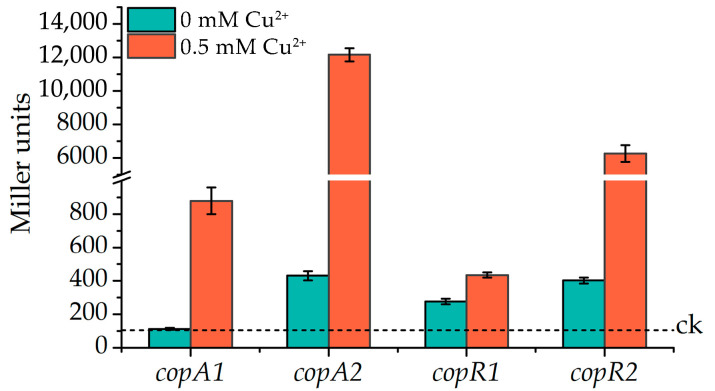
Copper-induced expression of *cop* genes. *β*-Galactosidase activity from *lacZ* fused to the *cop* promoters was measured in *P. putida* KT2440 after 4 h treatment with CuSO_4_. Control (ck): background activity measured in cells carrying the empty reporter vector. The data represent the mean ± standard deviation of three replicates.

To investigate the expression patterns of the *copAB* and *copRS* clusters, DNA fragments containing each promoter region were fused to the *β*-galactosidase gene *lacZ*, and the resulting reporter plasmids were subsequently introduced into *P. putida* KT2440 strains. *β*-galactosidase activity was measured to assess promoter activity after treatment with 0.5 mM Cu^2+^. In the control group without copper, the *copAB1* promoter in the wild-type strain remained transcriptionally inactive, as evidenced by undetectable levels of *β*-galactosidase activity. In contrast, the promoters of *copAB2*, *copRS1*, and *copRS2* exhibited basal levels of expression (Figure 4). Upon exposure to 0.5 mM Cu^2+^ for 4 h, all four promoters were significantly induced, with the *copAB2* and *copRS2* promoters showing markedly higher expression activity (>10-fold) compared to the *copAB1* and *copRS1* promoters (Figure 4).

**Figure 5:**
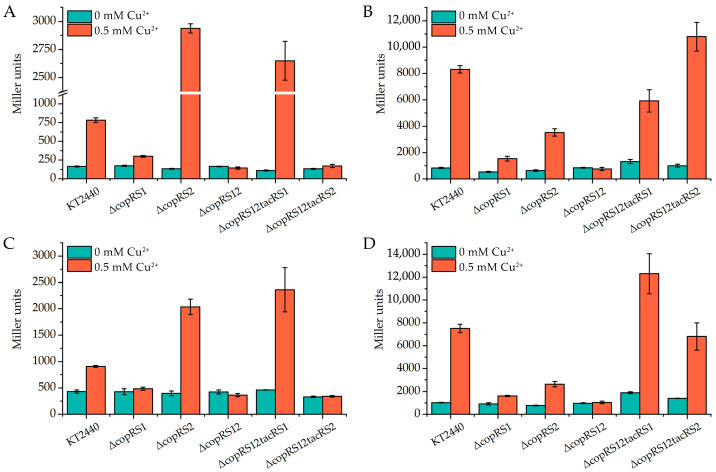
Regulation of *cop* genes by CopRS1 and CopRS2. *β*-Galactosidase activity from *lacZ* fused to the promoters of *copA1* (**A**), *copA2* (**B**), *copR1* (**C**), and *copR2* (**D**) was measured in *P. putida* strains after treatment with CuSO_4_. For complementation, plasmids pB403-*copRS*1 (tacRS1) and pB403-*copRS*2 (tacRS2) were introduced into Δ*copRS12*. The data represent the mean ± standard deviation of three replicates.

The expression of *copAB1* and *copRS1* showed consistent behavior in that both gene clusters failed to respond to copper induction in Δ*copRS1* and Δ*copRS12*. Notably, complementation of Δ*copRS12* with *copRS1* but not *copRS2* restored copper-responsive activation of these promoters (Figure 5A,C), establishing CopRS1 as their primary regulator. Intriguingly, these promoters displayed hyperactivation in Δ*copRS2* under copper stress, and this enhanced response persisted in the Δ*copRS12* strain complemented with *tac* promoter-driven *copRS1* (Figure 5A,C). These observations suggest that CopRS2 exerts a repressive effect on *copAB1* and *copRS1* expression.

For *copAB2* and *copRS2*, similar regulatory patterns were observed. Individual deletion of either *copRS1* or *copRS2* severely attenuated their expression under copper challenge, while dual deletion (Δ*copRS12*) completely abolished the copper-dependent activation (Figure 5B,D). Furthermore, introduction of either *copRS1* or *copRS2* under the control of the *tac* promoter into Δ*copRS12* restored promoter activity for both clusters (Figure 5B,D), indicating that both CopRS systems contribute to activation of *copAB2* and *copRS2*. However, functional divergence was also observed in that CopRS2 activated *copAB2* more strongly but *copRS2* more weakly than CopRS1 (Figure 5B,D). This indicates a difference in gene activation ability between these two CopRS two-component systems.

### 2.4. Identification of the Transcription Start Sites of Cop Genes

**Figure 6:**
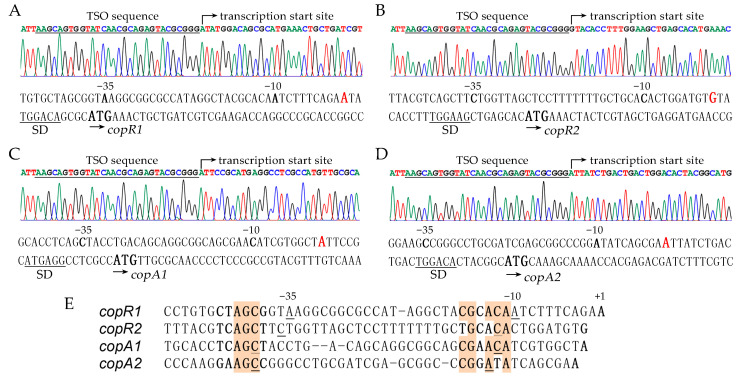
Identification of *cop* gene promoters. Transcription start sites of *copRS*1 (**A**), *copRS*2 (**B**), *copAB*1 (**C**), and *copAB*2 (**D**) identified by 5′-RACE are marked in red. (**E**) Analysis of sequences near −10 and −35 sites in *cop* promoters.

To elucidate the transcriptional regulation of *copAB* and *copRS* clusters, we mapped their transcription start sites using 5′ rapid amplification of cDNA ends (5′-RACE) assays. Prior to RNA extraction, *P. putida* KT2440 cells were treated with 0.5 mM Cu^2+^ for 2 h to induce optimal expression of copper-responsive genes. The analysis revealed transcription start sites located 13 bp, 23 bp, 20 bp, and 27 bp upstream of the initiation codons of *copR1*, *copR2*, *copA1*, and *copA2*, respectively (Figure 6A–D). No typical −10/−35 boxes recognized by major sigma factors (RpoD, RpoS, RpoN, RpoH, and FliA) were identified upstream of these sites. In *Myxococcus xanthus*, the extracytoplasmic function (ECF) sigma factor CorE was found to regulate transcription of the multicopper oxidase gene *cuoB* and two P1B-type ATPase genes (named *copA* and *copB* in *M*. *xanthus*) [41]. However, no CorE homolog was present among the 19 known ECF sigma factors in *P. putida* KT2440, and no features of CorE-dependent promoters were observed within the *cop* promoter regions [42]. According to the alignment analysis, motifs resembling “CGCACA” and boxes containing “AGC” were found near −10 and −35 positions, respectively, in all four promoters (Figure 6E). These findings suggest copper-responsive transcription in this strain might involve unidentified sigma factors or transcription mechanisms.

### 2.5. DNA Binding of CopR1 and CopR2 to the Cop Promoters

**Figure 7:**
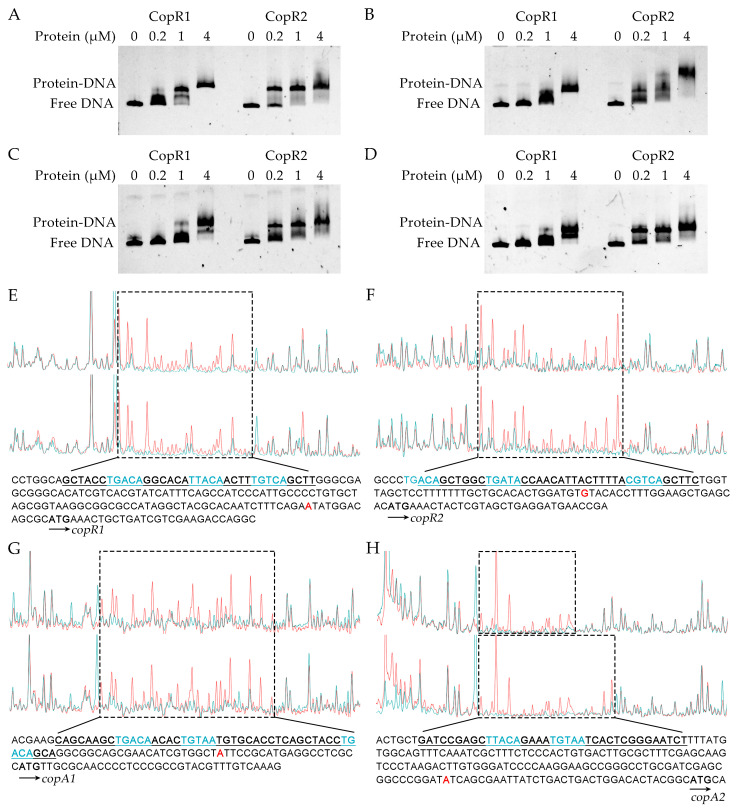
Identification of CopR binding sites in *cop* promoters. (**A**–**D**) Binding affinity of CopR1 and CopR2 to the promoters of *copRS1* (**A**), *copRS2* (**B**), *copAB1* (**C**), and *copAB2* (**D**). (**E**–**H**) DNase I footprinting analysis of CopR1 and CopR2 binding in the *copAB1* (**E**), *copAB2* (**F**), *copRS1* (**G**), and *copRS2* (**H**) promoters. Blue peaks represent samples incubated with CopR1 (upper panels) or CopR2 (lower panels); red peaks represent controls without CopR proteins. Underlined sequences represent regions protected by CopR. Blue-highlighted motifs denote sequences similar to “TGACA” or “TGTCA”. Red nucleotides indicate the transcription start sites.

The response regulators CopR1 and CopR2 were predicted to possess an N-terminal receiver domain and a C-terminal DNA-binding domain [43]. Phosphorylation of the conserved aspartyl residue (Asp51 in both proteins; Figure S1C) within CopR homodimers enables activation of target gene transcription [35]. To determine the binding affinity of CopR regulators for the *cop* promoters, electrophoretic mobility shift assays (EMSAs) were conducted using purified CopR proteins and 6-Carboxyfluorescein (6-FAM)-labeled promoter fragments spanning key regulatory regions (−229 to +29 bp for *copR1*, −237 to +31 bp for *copR2*, −202 to +37 bp for *copA1*, and −222 to +44 bp for *copA2*, relative to their translation start sites). Because phosphorylated CopR exhibits higher DNA-binding affinity [31], the regulators were pre-incubated with the phosphoryl donor carbamyl phosphate prior to contact with DNA probes. As shown in Figure 7A–D, both CopR1 and CopR2 reduced the electrophoretic mobility of all promoter probes, indicating binding affinity for all four *cop* promoters.

CopR-binding sites in *cop* promoters were mapped by DNase I footprinting analysis. Both regulators protected specific regions of each *cop* promoter from DNase I cleavage, with protected sequences spanning positions −144 to −110 bp, −96 to −58 bp, and −89 to −43 bp relative to the initiation codons of *copR1*, *copR2*, and *copA1*, respectively (Figure 7E–G). For *copA2*, CopR1 protected nucleotides −185 to −159 bp upstream, whereas CopR2 protected a longer region from −185 to −149 bp (Figure 7H). Previous studies have reported that *P. aeruginosa* CopR and *E. coli* CusR bind to DNA sequences containing inverted repeats with the consensus “TGACA-N4-TGTCA” [28,44]. Correspondingly, CopR1- and CopR2-binding sequences harbor similar motifs. CopR monomers likely interact with half-sites resembling “TGACA”, and multiple such motifs appear within these protected regions, explaining the extended CopR-binding regions in *cop* promoters (Figure 7E–H).

## 3. Discussion

According to genome annotation, *P. putida* KT2440 possesses two *copAB* and two *copRS* clusters [43]. CopA1 and CopB1 exhibit a high degree of amino acid sequence homology to CopA2 and CopB2, respectively; however, their corresponding gene clusters show divergent GC content (66.41% for *copAB1*, 57.08% for *copAB2*). A previous study speculated that at least one *copAB* cluster was acquired via horizontal gene transfer [38]. A similar GC content disparity exists between *copRS1* (66.45%) and *copRS2* (53.60%), further supporting the potential horizontal acquisition of these clusters from distinct genetic sources. The retention of these *copAB* and *copRS* clusters in *P. putida* KT2440 raises the question of functional differentiation between them. In this study, we investigated the roles of these *cop* gene clusters in copper resistance.

Our results reveal that both *copAB1* and *copAB2* constitute functional copper resistance genes. However, *copAB1* contributes substantially less to copper resistance than *copAB2*, as its detoxification capacity was only detectable in a *copAB2*-null background (Figure 2E,F). This observation aligns with a previous report showing greater copper sensitivity in the *copA2* mutant compared to *copA1* mutant [45]. Promoter activity assays revealed that *copAB1* was induced by copper stress, but its expression level remained significantly lower than *copAB2* (Figure 4). This low expression partially explains its limited protective role. This was also supported by the greater copper resistance exhibited by the Δ*copAB12* strain expressing *copAB1* from the high-activity *tac* promoter compared to its native promoter (Figure 2E,F). In contrast, *copAB2* maintained relatively high basal transcription regardless of copper stress, indicating roles in both copper homeostasis and acute detoxification. The significantly stronger copper-responsive induction of *copAB2* further establishes CopAB2 as the primary copper resistance determinant. Although CopAB1 appears functionally redundant in copper resistance, *P. putida* has retained this gene cluster, suggesting potential alternative physiological roles. Actually, CopA was also predicted to be a multi-copper laccase that showed oxidase activity toward lignin model compounds and polymeric lignin [45]. However, the differences in additional functions between these two gene clusters remain to be explored. Beyond the CopAB systems, *P. putida* KT2440 encodes the RND efflux pump CusCBA, which mediates copper extrusion [38]. Furthermore, biofilm formation confers protection against copper toxicity via adsorption of ionic copper by extracellular polymeric substances (EPS) [46]. Notably, EPS-associated extracellular DNA exhibits affinity for copper ions and facilitates their conversion into species resembling copper phosphate [47]. High copper levels also promote chemotaxis by relieving the inhibition of CheA autophosphorylation imposed by the copper-binding protein CosR in *P. putida*, and this negative chemotactic response may enable bacterial escape from copper stress [48,49]. Collectively, these adaptive mechanisms significantly mitigate the cytotoxicity from environmental copper.

Elevated copper levels also induced expression of the two-component systems CopRS1 and CopRS2. Expression of *copRS1* coincided with that of *copAB1*, while *copRS2* expression paralleled *copAB2* (Figure 5A–D). This aligns with the established role of CopRS systems as conserved copper-responsive regulators of *cop* operons in bacteria [31,50,51]. In *P. putida* KT2440, both CopRS1 and CopRS2 were essential for full copper-induced activation of the *cop* promoters (Figure 5A–D). CopR1 and CopR2 bind directly to all four *cop* promoters, specifically targeting regions containing the conserved “TGACA” motif (Figure 7E–H), which is also present in CopR-binding sequences from other bacteria [28,44]. This shared binding specificity suggests potential competition between these two regulators for promoter occupancy. Intriguingly, CopR1 activated all four *cop* promoters under copper stress, whereas CopR2 selectively enhanced *copRS2* and *copAB2* expression but failed to activate *copRS1* and *copAB1* (Figure 5A–D). Unidentified factors might contribute to these differential outcomes. Specifically, competitive binding of CopR2 to the *copAB1* and *copRS1* promoters might attenuate CopR1-mediated activation. This provides a mechanistic explanation for the significantly higher expression of *copAB1* and *copRS1* observed in *copRS2*-null strains compared to the wild-type (Figure 5A,C). Given that CopR1 activates *copRS2* more potently than CopR2, a feedback regulatory loop likely exists between the two CopRS systems. In this loop, CopR1 efficiently activates *copRS2* expression, while elevated CopR2 levels suppress *copRS1* expression, thereby preventing excessive CopR1-dependent activation of *copRS2* and further constraining CopAB expression.

## 4. Materials and Methods

### 4.1. Bacterial Strains and Growth Conditions

The bacterial strains and plasmids used in this study are listed in Table S2. All strains were incubated in LB medium under shaking at 180 r/min. *P. putida* KT2440 and its derivatives were grown at 30 °C, while the *E. coli* strains were grown at 37 °C. When required, the following additives were used in culture media at these concentrations: chloramphenicol, 25 μg/mL; gentamycin, 20 μg/mL; tetracycline, 30 μg/mL; kanamycin, 50 μg/mL; and isopropyl *β*-D-thiogalactoside (IPTG), 0.5 mM.

### 4.2. Construction of P. putida Mutants and Complemented Strains

All primers for plasmid construction are listed in Table S3. To knock out *copAB1*, *copAB2*, *copRS1*, and *copRS2* gene clusters, flanking regions of each gene cluster were amplified by polymerase chain reaction (PCR) using primers for mutant construction and cloned into SacI-digested pDS3.0 using the ClonExpress II one-step cloning kit (Vazyme). The resulting plasmids harbored in *E. coli* S17-1 were conjugated into *P. putida* KT2440 for allelic exchange. The *sacB* counter-selection gene and sucrose were used for mutant selection as described previously [52]. For complementation, promoter-containing *cop* fragments were cloned into XbaI/BamHI-digested pBBR1MCS-5 [53]. Promoter-less *cop* fragments were cloned into the EcoRI/BamHI-digested pBBR1-403 [54], which contains a *tac* promoter, to generate inducible expression plasmids (Table S2). These plasmids were transferred from *E. coli* S17-1 to *P. putida* strains by conjugation.

### 4.3. Growth Assay in the Presence of Cu^2+^

Overnight cultures of *P. putida* strains were diluted in fresh LB medium supplemented with the indicated CuSO_4_ concentration to an optical density at 600 nm (OD_600_) of 0.05. A volume of 150 μL of the diluted bacterial cultures was transferred to a 96-well plate. The plate was incubated at 30 °C in a synergy H1 microplate reader (BioTek, Winooski, VT, USA), and the absorbance at 600 nm (A_600_) of the cultures was continuously monitored at 30 min intervals.

### 4.4. Construction of Reporter Plasmids and β-Galactosidase Activity Measurement

Promoter regions of *cop* genes were amplified from *P. putida* KT2440 chromosomal DNA using primers for promoter amplification (Table S3) and inserted into XbaI/PstI-digested pBRTZ reporter vector [55], yielding pBRTZ-*copA1*, pBRTZ-*copA2*, pBRTZ-*copR1*, and pBRTZ-*copR2*. The resulting plasmids were transferred into *P. putida* KT2440 and its derivatives. Strains harboring reporter plasmids were pre-incubated in LB medium to an OD_600_ of 0.5 and then treated with 0.5 mM CuSO_4_. After 4 h incubation, cultures were sampled for *β*-galactosidase assays. Reaction mixtures contained 100 µL bacterial culture samples, 400 µL Z buffer (60 mM Na_2_HPO_4_, 40 mM NaH_2_PO_4_, 10 mM KCl, 1 mM MgSO_4_, and 50 mM *β*-mercaptoethanol), 25 µL 0.1% (*w*/*v*) sodium dodecyl sulfate (SDS), 50 µL chloroform, and 100 µL 0.4% (*w*/*v*) 2-nitrophenyl-*β*-D-galactopyranoside (ONPG). Reactions were stopped with 250 µL 1 M Na_2_CO_3_ when yellow color appeared, and reaction time was recorded. After centrifugation, absorbance at 420 nm (A_420_) of the supernatant was measured. *β*-Galactosidase activity was calculated as

Miller units = 1000 × A_420_/(A_600_
× V × T)

where A_600_ = absorbance at 600 nm of the bacterial culture; V = volume of bacterial sample (mL); T = reaction time (min).

### 4.5. Identification of Transcription Start Site

Transcription start sites were mapped using 5′-RACE assay as described previously [56,57]. Oligonucleotides for 5′-RACE are listed in Table S3. Briefly, total RNA was isolated from the *P. putida* KT2440 cultures treated with 0.5 mM CuSO_4_ for 2 h using Trizol reagent (Vazyme, Beijing, China). A 5′ cap structure was added to RNA by treatment with vaccinia capping enzyme (New England Biolabs, Ipswich, MA, USA). After removing residual genomic DNA with DNase I treatment, the capped RNA was purified via ethanol precipitation. The purified RNA was used as template for cDNA synthesis. Following the first round of reverse-transcription reaction, template-switch oligo (TSO) RNA was added to the reaction system to enable template switching during the next round of cDNA synthesis, thereby adding the TSO sequence to the cDNA. The resulting cDNA was amplified by PCR with gene-specific 5′-RACE primers, and the products were cloned into pMD19-T vector (Takara, Osaka, Japan). Finally, transcription start sites of the target genes were identified by sequencing the cloned inserts.

### 4.6. Purification of CopR1 and CopR2

The coding sequences of *copR1* and *copR2* were amplified and ligated into NcoI/XhoI-digested pET28a, generating pET28a-*copR1* and pET28a-*copR2*. *E. coli* BL21 (DE3) harboring these plasmids were incubated in LB medium to an OD_600_ of 0.5, then induced with 0.5 mM IPTG at 16 °C for 6 h. Cells were pelleted and resuspended in lysis buffer (50 mM NaCl, 10% (*v*/*v*) glycerol, 4 M urea, and 10 mM Tris-HCl; pH 8.0), and lysed using a cell disruptor. His_6_-tagged proteins in the supernatant were purified by Ni-NTA spin column and eluted with E250 buffer (250 mM NaCl, 10% (*v*/*v*) glycerol, 250 mM imidazole, and 10 mM Tris-HCl; pH 8.0).

### 4.7. EMSAs

Binding affinity of CopR regulators for *cop* promoters was detected as described previously [57]. The 6-FAM-labeled promoter probes were synthesized in two PCR rounds using primers listed in Table S3. The first PCR amplified the fragments containing promoter regions, and the second PCR added the 6-FAM tag to DNA fragments using 6-FAM-labeled primer FAM-M13F. Binding reactions (20 μL) contained 20 nM DNA probe, 10 mM Tris-HCl (pH 7.5), 50 mM KCl, 10 mM MgCl_2_, 5% (*v*/*v*) glycerol, and phosphorylated CopR1 or CopR2, which were pre-incubated with 50 mM carbamyl phosphate for 30 min. After incubation for 20 min at 4 °C, 15 μL of each mixture was loaded on a 5% (*w*/*v*) non-denaturing polyacrylamide gel. Electrophoresis was performed in TGE buffer (12.5 mM Tris-HCl, 96 mM glycine, and 2.5 mM EDTA-2Na; pH 8.3) on ice at 100 V for 90 min. The fluorescence of DNA probes was detected using a ChemiDoc XRS+ (BioRad, Hercules, CA, USA).

### 4.8. DNase I Footprinting Assay

The binding sites of CopR in promoters were mapped by DNase I footprinting. Briefly, 40 nM 6-FAM-labeled DNA probes were incubated with 5 μM phosphorylated CopR or bovine serum albumin (control) in a binding system as described in Section 4.7. The samples were treated with 1.5 U/mL DNase I and 20 mM MgCl_2_ for 2 to 5 min at room temperature. Then, 200 μL samples were placed at 1 min intervals into new centrifuge tubes, and then, the digestion reaction was terminated by denaturing DNase I with 100 µL phenol-chloroform (1:1, *v*/*v*) and 80 °C heating for 2 min. The DNA was purified by alcohol precipitation, and the final samples were analyzed by DNA Sequencer. Peak signals were processed with Peak Scanner Software v1.0 (Applied Biosystems, Waltham, MA, USA).

## 5. Conclusions

**Figure 8:**
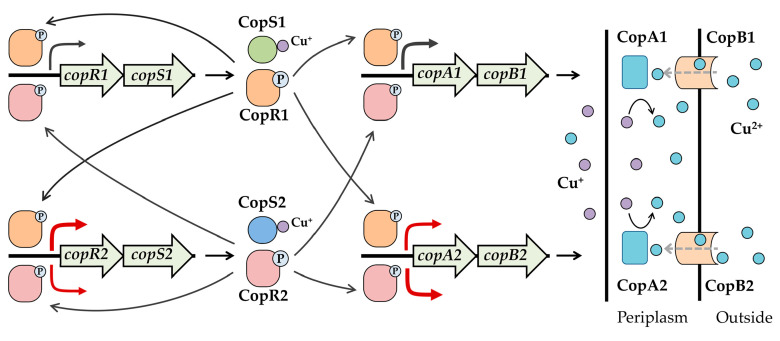
Proposed model for *cop* gene regulation by CopRS. CopR1 and CopR2 compete for binding to the four *cop* promoters. CopR1 effectively activates transcription of all four genes, while CopR2 activates only *copRS2* and *copAB2*. Compared to *copAB1*, high expression of *copAB2* confers stronger copper resistance to the strain.

This study has shown that the two *copAB* gene clusters in *P. putida* function in copper resistance. However, *copAB2* plays a more critical role, which may be partially attributed to its significantly higher expression activity than that of *copAB1*. Although CopR1 and CopR2 bind directly to the same region in the promoters of *copAB1*, *copAB2*, *copRS1*, and *copRS2*, only CopR1 activates all four promoters. In contrast, CopR2 suppresses activation of *copAB1* and *copRS1* while inducing *copAB2* and *copRS2* (Figure 8). Through coordinated regulation by the two CopRS systems, CopAB proteins may be expressed at appropriate levels to maintain copper homeostasis in the periplasm.

## Data Availability

The original contributions presented in this study are included in the article. Further inquiries can be directed to the corresponding author.

## Supplementary Materials

The following supporting information can be downloaded at https://www.mdpi.com/article/10.3390/ijms26178172/s1. Refs [37,53,54] are cited in Supplementary Materials.
