# Supplementary material for: Regulatory Characterization of Two Cop Systems for Copper Resistance in Pseudomonas putida
Source: Int J Mol Sci. 2025 Aug 22;26(17):8172. doi: 10.3390/ijms26178172 (PMC12428095; doi:10.3390/ijms26178172)
Supplement: Supplementary file 1 [file ijms-26-08172-s001.zip › ijms-3791473-supplementary.pdf]

Supplementary materials

Table S1. Amino acid sequence identities of *CopA* and *CopB* homologs.

P. putida KT2440	PcoA (Q47452) (*E. coli*)	PcoA (Q9I250) (*P. aeruginosa*)	*P. putida* KT2440	PcoB (Q47453) (*E. coli*)	PcoB (Q9I251) (*P. aeruginosa*)
CopA1	64.85%	68.91%	CopB1	47.57%	55.37%
CopA2	68.91%	76.46%	CopB2	59.41%	66.18%

Sequences of PcoA/PcoB can be obtained from UniProtKB using the accession number.

Table S2. Bacterial strains and plasmids used in this study.

**Strains and plasmids**	**Description**	**Reference**
**Strains:**		
E. coli S17-1	*RP4*$^-$, *mob*$^+$, *λpir*, host for plasmid construction	Lab stock
E. coli BL21(DE3)	*F*$^-$, *ompT*, *hsdS* (*rB*$^-$ *mB*$^-$), *gal*, *dcm* (DE3)	Lab stock
P. putida KT2440	Wild-type *Pseudomonas putida* KT2440, Cm^R	[1]
Δ*copAB1*	*copAB1* mutant of *P. putida* KT2440	This study
Δ*copAB2*	*copAB2* mutant of *P. putida* KT2440	This study
Δ*copAB12*	*copAB1-copAB2* double mutant of *P. putida* KT2440	This study
Δ*copRS1*	*copRS1* mutant of *P. putida* KT2440	This study
Δ*copRS2*	*copRS2* mutant of *P. putida* KT2440	This study
Δ*copRS12*	*copRS1-copRS2* double mutant of *P. putida* KT2440	This study
**Plasmids:**		
pDS3.0	Suicide vector, Gm^R, *R6K ori*, *sacB*	[2]
pDS-*copAB1*	Knockout vector for *copAB1*	This study
pDS-*copAB2*	Knockout vector for *copAB2*	This study
pDS-*copRS1*	Knockout vector for *copRS1*	This study
pDS-*copRS2*	Knockout vector for *copRS2*	This study
pBBR1MCS-5	Expression vector, Gm^R	[3]
pBBR1-*copAB1*	pBBR1MCS-5 carrying *copAB1* with its promoter	This study
pBBR1-*copAB2*	pBBR1MCS-5 carrying *copAB2* with its promoter	This study
pBBR1-*copRS1*	pBBR1MCS-5 carrying *copRS1* with its promoter	This study
pBBR1-*copRS2*	pBBR1MCS-5 carrying *copRS2* with its promoter	This study
pBBR1-403	Expression vector, Gm^R, *lacI*, *tac* promoter	[4]
pB403-*copAB1*	pBBR1-403 carrying *copAB1*	This study
pB403-*copAB2*	pBBR1-403 carrying *copAB2*	This study
pB403-*copRS1*	pBBR1-403 carrying *copRS1*	This study
pB403-*copRS2*	pBBR1-403 carrying *copRS2*	This study
pBRTZ	Reporter vector, Tet^R, promoter-less *lacZ*	[5]
pBRTZ-*copA1*	pBRTZ carrying *copAB1* promoter	This study
pBRTZ-*copA2*	pBRTZ carrying *copAB2* promoter	This study
pBRTZ-*copR1*	pBRTZ carrying *copRS1* promoter	This study
pBRTZ-*copR2*	pBRTZ carrying *copRS2* promoter	This study

pET28a	Expression vector, Km^R, *lacI*, *T7* promoter	Lab stock
pET28a-*copR1*	pET28a carrying *copR1*	This study
pET28a-*copR2*	pET28a carrying *copR2*	This study

Cm^R, chloramphenicol resistance; Gm^R, gentamycin resistance; Tet^R, tetracycline resistance; Km^R, kanamycin resistance.

Table S3. Primers used in this study.

Primers	sequence
**Primers for mutant construction**	
copAB1upS	AGGTACCGCATGCGATATCGAGCTCGGCAACCTCGAATCCCTGG
copAB1upA	GTTGTTGACGGTCAGTGCG
copAB1dnS	CGCACTGACCGTCAACAACGAACAAGGTGCGGGGCTGG
copAB1dnA	TTTGTGGAATTCCCGGGAGAGCTCTGGCGACCACGGTGTCCTG
copAB2upS	AGGTACCGCATGCGATATCGAGCTCCGGAACTGTAATGCCGAGC
copAB2upA	ATCAAAATCGGTGCCTGTCA
copAB2dnS	TGACAGGCACCGATTTTGATGCCACAGCGTTCATCGGAG
copAB2dnA	TTTGTGGAATTCCCGGGAGAGCTCTTGGCAAGATTGGGCGGTG
copRS1upS	AGGTACCGCATGCGATATCGAGCTCGCCTTGTCGCCCAGGTTGA
copRS1upA	ATCAGCAGGTCGTGGTCGC
copRS1dnS	GCGACCACGACCTGCTGATCGGCAGGAGGACAGATCAAG
copRS1dnA	TTTGTGGAATTCCCGGGAGAGCTCCATTGCCATCCTGCGTACCT
copRS2upS	AGGTACCGCATGCGATATCGAGCTCTTTTCCGCCTTCACCAGAAC
copRS2upA	CTTCCCATCCTTCTAGCCCC
copRS2dnS	GGGGCTAGAAGGATGGGAAGGGCAACCATTGACCCACAAC
copRS2dnA	TTTGTGGAATTCCCGGGAGAGCTC GGCTCTGACCGTAGGTATCG
**Primers for promoter amplification**	
copA1pS	CACCGCGGTGGCGGCCGCTCTAGAGGGCGACCATTTGGAACTG
copA1pA	TGAATGAGATTTAGTCATCTGCAGTTTGACAAACGTACGGCGG
copA2pS	CACCGCGGTGGCGGCCGCTCTAGAATCACTTTTGGAATGGCTGG
copA2pA	TGAATGAGATTTAGTCATCTGCAGGACGAAAGATCGTCTCGTGG
copR1pS	CACCGCGGTGGCGGCCGCTCTAGAGCCAAAGGCGAAGCTCTGC
copR1pA	TGAATGAGATTTAGTCATCTGCAGGGCCTGGTCTTCGACGATCA
copR2pS	CACCGCGGTGGCGGCCGCTCTAGAGGTTTTGGGGTGATCTTGGT
copR2pA	TGAATGAGATTTAGTCATCTGCAGCGGTTCATCCTCAGCTACGA
**Primers for gene cloning** (used with the sense strand primers for promoter amplification)	
copAB1A	GGGGATCCGCCAGCAGGCTCAGGGTCA
copAB2A	GGGGATCCGAAATACGCGGCCCCCAAT
copRS1A	GGGGATCCGACCATTTACCCGGCCTAC
copRS2A	GGGGATCCATGGTTAGCAGTCGTAGGAAT
**Primers for inducible expression plasmids**	
copAB1Stac	CAATTTCACACAGGAAACAGAATTCATGTTGCGCAACCCCTCCC
copAB1Atac	CCTGCAGGTCGACTCTAGAGGATCCGCCAGCAGGCTCAGGGTCA

copAB2Stac	CAATTTCACACAGGAAACAGAATTCATGCAAAGCAAAACCACGA
copAB2Atac	CCTGCAGGTCGACTCTAGAGGATCCGAAATACGCGGCCCCCAAT
copRS1Stac	CAATTTCACACAGGAAACAGAATTCATGAAACTGCTGATCGTCGA
copRS1Atac	CCTGCAGGTCGACTCTAGAGGATCCGACCATTTACCCGGCCTAC
copRS2Stac	CAATTTCACACAGGAAACAGAATTCATGAAACTACTCGTAGCTGAGG
copRS2Atac	CCTGCAGGTCGACTCTAGAGGATCCATGGTTAGCAGTCGTAGGAAT
**Oligonucleotides for 5′-RACE**	
copR1-RC	CATCACGTCGAGGATCAGCAGGTCG
copR2-RC	CCATCCTTCTAGCCCCGGCATCATC
copA1-RC	TCGGCCTGTGATGTTGACCGGAGTC
copA2-RC	TGCCTGTCAGAACATTCGGTTGGCC
SMARTerIIA	AAGCAGTGGTATCAACGCAGAGTACGCGGG
TSO-RNA	AAGCAGUGGUAUCAACGCAGAGUACGCGGG (ssRNA)
**Primers for protein purification**	
28a-copR1S	ACTTTAAGAAGGAGATATACCATGAAACTGCTGATCGTCGAA
28a-copR1A	TGGTGGTGGTGGTGGTGCTCGAGCTCGGTGCGCTCTTCGAGTA
28a-copR2S	ACTTTAAGAAGGAGATATACCATGAAACTACTCGTAGCTGAGG
28a-copR2A	TGGTGGTGGTGGTGGTGCTCGAGCTCCGGTGCATCCATCATGT
**Primers for 6-FAM DNA probe synthesis**	
M13-copA1pS	TGTAAAACGACGGCCAGTAGCAGCACGCCATCGAGGT
copA1pA	CTTTGACAAACGTACGGCGG
M13-copA2pS	TGTAAAACGACGGCCAGTAGCCAGAGCCATAAACACGG
copA2pA	GCCAGGCCTTTGACGAAAGA
M13-copR1pS	TGTAAAACGACGGCCAGTGGGTGGCGGTAATGAACAGG
copR1pA	GCCTGGTCTTCGACGATCAG
M13-copR2pS	TGTAAAACGACGGCCAGTCAGCAGCATCGATGTAGCCA
copR2pA	TCGGTTCATCCTCAGCTACG
FAM-M13F	6FAM-TGTAAAACGACGGCCAGT

```
A CopA1    1 MLRNPSRRTFVKGLGAASTLAGLGLWRPLVQA----AEGRDLAGQHFELFIGQTPVNITGRPRTALTVNNSLPGPL  72
  CopA2    1 MQSKTTRRSFVKGLAATGLLGGLGMWRAPVWAVTSPGQPNVLTGTDFDLYIGELPVNITGTVRTAMAINGSIPGPI  76

  CopA1   73 LRWREGDTVTLRVRNRLAQDTSIHWHGIILPANMDGVPGLSFAGIEPGGDYLYQFTLRQSGTYWYHSHSGLQEQAG 148
  CopA2   77 LRWREGDTVTLRVRNRLQQDTSIHWHGIILPANMDGVPGLSFHGIAPDGMYEYKFKVQQNGTYWYHSHSGFQEQVG 152

  CopA1  149 VYGAIVIEPREPETHRYQRDHVLLFSDWSDQAPEHLMATLKTQSDAYNFHKRTVGDFIDDVAENGWSATVAERTAW 224
  CopA2  153 VYGALVIDAKEPEPFTYDRDYVVMLSDWTDEDPARVLSKLKKQSDYYNYHKRTVGDFVNDVSEMGWSAAVADRKMW 228

  CopA1  225 ARMRMSPTDLADISAATYTYLLNGQPPQGNFTCLFQPGETVRLRLINASAMTYFDFRIPGLKLTVIAADGLPVTPV 300
  CopA2  229 AEMKMSPTDLADVSGYTYTYLMNGQAPDGNWTGVFKPGEKIRLRFINGSAMTYFDVRIPGLKMTVVAADGQHVKRV 304

  CopA1  301 SVDELRIAVAETYDVLVTVGDQPAYTLFAQSMDRTGFARGTLARAAGLQAPVPAPDPRPVLSMEDMG--HG---GM 367
  CopA2  305 AVDEFRIAVAETYDVIVEPEDEQAYTIFAQSMDRTGYSRGTLAVREGMQAAVPAVDPRPLISMSDMGMDHGSMAGM 380

  CopA1  368 D----------------------------------AMAGMDHRNPQGMNHGSMAGMDHAAMS-------------- 399
  CopA2  381 DHGNMAGMDHSKMAGMDHGNMAGMDHSKMAGMDHGNMTGMDHSKMAGMDHGSMAGMDHSKMAEMDHGGMAGMDHSK 456

  CopA1  400 --------------------------------------AMQQHPISETDNPLVDMQTMAPRPNLADPGIGLRDNGR 437
  CopA2  457 MAGMDQGGMADMDHSKMAGMDQGGMADMDHSSMEGMGGAMQSHPASETNNPLVDMQTMSPTPKLDDPGIGLRNNGR 532

  CopA1  438 RVLTYADLRSPYPDPDGRPPSRDIELHLTGHMERFAWSFDGIKFSDAEPLRLTYGERVRITLVNDTMMTHPIHLHG 513
  CopA2  533 RVLTYADLRSTFIDPDGREPGRTIELHLTGHMEKFAWSFDGVKFSDAEPLRLKYGERLRITLVNDTMMTHPIHLHG 608

  CopA1  514 MWSDLEDEHGQFLVRKHTVDIPPGSRRTYRVTADALGRWAYHCHLLYHMETGMLREVRVDE 574
  CopA2  609 MWSDLEDEDGNFMVRKHTIDMPPGSKRSYRVTADALGRWAYHCHLLFHMEMGMFREVRVDE 669

B CopB1    1 MNDTRNR--------SF-VAGVALLTLLASER---AMAAGSE---------------------------------- 30
  CopB2    1 MTNSLARPSLLALTVSFSMLGAAAPSFAAEEMDHSAMGHGAMEMDSAPSADQMPGMDHAQMGASKQQKKPAPVDHS 76

  CopB1   31 ------------HTNHGQMGHQAM------PINHESMGHDQPSPSQ-PRTPLPVITDADRRAAFPPLPGHQAHDRA 87
  CopB2   77 KMKHSQPVAKPAHMDHSKMDHGSMQGMDHGAMDHSMMNHGSDAPTTTSRTPIPVLTDADRQAAFPPLGGHQMHDSA 152

  CopB1   88 LNWAVIVDKLEYQNFESSSALNWNATAWVGGDIDRLWLRTEGEREQGKTHKAELQALWGHAVSPWWELVAGARQDF 163
  CopB2  153 INSFFLLDKLEYQDADEGSALAWEASGWVGGDINRLWIRSEGERTNGVTEDAELQLLYGRSISPWWDVVAGVRQDF 228

  CopB1  164 KPASGQTWAAFGIQGTPLYGLELQATAYAGERQQTALRLEAAYAMLLTNRWILEPTLEANLYGRNDAGREQGAGLA 239
  CopB2  229 QPGSPQTWGAFGIQGMALYDFEAEATAFIGENGQTAARLEGEYDILLTNRLILQPTAEANFYGKNDPERGVGSGLA 304

  CopB1  240 DSEVGLRLRYEITRGFAPYVGLSFNRLHGSRANQAREDGEDLGQTRLVAGIRLRF 294
  CopB2  305 NTEVGLRLRYEIVRQFAPYIGVTWNRSYGKTADLIRDEGGDVDEARFVAGIRMWF 359

C CopR1    1 MKLLIVEDQARTGQYLSQGLSEAGFATELATDGETGQFLALTGDHDLLILDVMLPGRDGWQILQAVRQAGLDTPVL 76
  CopR2    1 MKLLVAEDEPKTGAYLQQGLAEAGFTVDRVLTGTDALQHALSESYDLLILDVMMPGLEGWEVLRMVRAAGKDVPVL 76

  CopR1   77 FLTARDAVEDRVHGLELGADDYLVKPFAFSELLARVRSLLRRGTSPSQDTILSLADLRLDLIRRRAERAGLRIDLT 152
  CopR2   77 FLTARDGVDDRVKGLELGADDYLIKPFAFSELLARVRTLLRRGTSTAAQTTMKMADLEVDLLKRRATRSGKRIDLT 152

  CopR1  153 AKEFSLLELLLRRQGEVLPKSLIASQVWDMNFDSDTNVIEVAIRRLRLKIDDPHPNKLIHTVRGMGYVLEERTE 226
  CopR2  153 AKEFSLLELLMRRRGEVLPKSLIASQVWDMNFDSDTNVIEVAIRRLRAKIDDDFAPKLIHTARGMGYMMD-APE 225

D CopS1    1 MM-RRVSLGSRLALLFAACTATVSLGAGLLFSRASEQHFVELDQQLLDSRLSLFRTQLAGVSTADELQARLPALRD 75
  CopS2    1 MILTRSSLVKRLTLMIMFAVIAVLVVAGISFNMLSQHHFRMLDEQALSEKLESTRHILSIQAPGTGMEELRPQLRA 76

  CopS1   76 ELSHQADLALRISASNGATWFESRSG--LPHA---AQATGLATLHAPGIDYRSLSVPL-TQGAMQSPRLTLYLDIT 145
  CopS2   77 LLGAHQDLTAEILTSEGEVLFSDLKAVQIPDKYKRADKEEMWEWQDESHNFRGITTDVEIQGQPAPGTVMLMLDIT 152

  CopS1  146 HHQHFLQGMQRLIWLTVGL--SALITALLGAWAARSGLRPLRQMGQVAASVSARSLTTRLPVAQMPEELAELASSM 219
  CopS2  153 SHAHFFETLQR—WFAIGLVISALVSAGIGWLVAKSGLRPVEQITKVATSISARSLQERIPLEPVPLELQTLILSF 226

  CopS1  220 NAMLQRLDDAFQRLSAFSADIAHELRTPLSNLLTHTQVTLTRPRSLEEYREALHGNLEELQWMAQMINDMLFLAKA 295
  CopS2  227 NGMLARLEDAFVRLSNFSADIAHELRTPVSNLLTHTEVVLTRKRDVDAYEENLYSNLEDLKRMSRMIDDMLFLAKS 302

  CopS1  296 DHGLLVPGDAPLALHDEVDALLEYYAPLAEDSDVQMLREGEAVLHGDQHMLRRALSNLLDNAMRFTPAGGQIKVTL 371
  CopS2  303 DNGLIIPEQVDIQLHDLVSKLFEYYQLLADDRGIRLTLQGKGVISGDRLMIDRAVSNILSNAVRYTPDGSGISVEI 378

  CopS1  372 ---GPGPTINVANTGLAIDPAALPRLFDRFYRVDPARREGSSEHAGLGLAITRSIVQAHGGCIRAECEGGWTRFVI 444
  CopS2  379 QQAADKVTLTIKNGGATIDPQHINKIFDRFYRADPARREGGPSNAGLGLAITRSIVKAHDGKVWCTSSEGVTAFNF 454

  CopS1  445 EFTQDR--------- 450
  CopS2  455 IFPAAHRTAHIRTRH 469
```

Figure S1. Amino acid sequence alignment of CopA (A), CopB (B), CopR (C), and CopS (D) homologues in *P. putida* KT2440. The MXXMXHXXM motifs are indicated by a line in panels A and B. The amino acid residues that accept phosphate group are marked in red in panels C and D.

References

1. Bagdasarian M., Lurz R., Ruckert B., Franklin F.C.H., Bagdasarian M.M., Frey J., Timmis K.N. Specific-purpose plasmid cloning vectors. II. Broad host range, high copy number, RSF1010-derived vectors, and a host-vector system for gene cloning in *Pseudomonas*. *Gene* **1981**, 16, 237-247.
2. Gao W., Liu Y., Giometti C.S., Tollaksen S.L., Khare T., Wu L., Klingeman D.M., Fields M.W., Zhou J. Knock-out of *SO1377* gene, which encodes the member of a conserved hypothetical bacterial protein family COG2268, results in alteration of iron metabolism, increased spontaneous mutation and hydrogen peroxide sensitivity in *Shewanella oneidensis* MR-1. *BMC Genomics* **2006**, 7, 76.
3. Kovach M.E., Elzer P.H., Hill D.S., Robertson G.T., Farris M.A., Roop R.M., 2nd, Peterson K.M. Four new derivatives of the broad-host-range cloning vector pBBR1MCS, carrying different antibiotic-resistance cassettes. *Gene* **1995**, 166, 175-176.
4. Nie H.L., Xiao Y.J., Liu H.Z., He J.Z., Chen W.L., Huang Q.Y. FleN and FleQ play a synergistic role in regulating *lapA* and *bcs* operons in *Pseudomonas putida* KT2440. *Env. Microbiol. Rep.* **2017**, 9, 571-580.
5. Liu H.Z., Xiao Y.J., Nie H.L., Huang Q.Y., Chen W.L. Influence of (p)ppGpp on biofilm regulation in *Pseudomonas putida* KT2440. *Microbiol. Res.* **2017**, 204, 1-8.
